# Role of Kinesin Heavy Chain in Crumbs Localization along the Rhabdomere Elongation in *Drosophila* Photoreceptor

**DOI:** 10.1371/journal.pone.0021218

**Published:** 2011-06-17

**Authors:** Garrett P. League, Sang-Chul Nam

**Affiliations:** Department of Biology, Baylor University, Waco, Texas, United States of America; Laboratoire Arago, France

## Abstract

**Background:**

Crumbs (Crb), a cell polarity gene, has been shown to provide a positional cue for the extension of the apical membrane domain, adherens junction (AJ), and rhabdomere along the growing proximal-distal axis during *Drosophila* photoreceptor morphogenesis. In developing *Drosophila* photoreceptors, a stabilized microtubule structure was discovered and its presence was linked to polarity protein localization. It was therefore hypothesized that the microtubules may provide trafficking routes for the polarity proteins during photoreceptor morphogenesis. This study has examined whether Kinesin heavy chain (Khc), a subunit of the microtubule-based motor Kinesin-1, is essential in polarity protein localization in developing photoreceptors.

**Methodology/Principal Findings:**

Because a genetic interaction was found between *crb* and *khc*, Crb localization was examined in the developing photoreceptors of *khc* mutants. *khc* was dispensable during early eye differentiation and development. However, *khc* mutant photoreceptors showed a range of abnormalities in the apical membrane domain depending on the position along the proximal-distal axis in pupal photoreceptors. The *khc* mutant showed a progressive mislocalization in the apical domain along the distal-proximal axis during rhabdomere elongation. The *khc* mutation also led to a similar progressive defect in the stabilized microtubule structures, strongly suggesting that Khc is essential for microtubule structure and Crb localization during distal to proximal rhabdomere elongation in pupal morphogenesis. This role of Khc in apical domain control was further supported by *khc*'s gain-of-function phenotype. Khc overexpression in photoreceptors caused disruption of the apical membrane domain and the stabilized microtubules in the developing photoreceptors.

**Conclusions/Significance:**

In summary, we examined the role of *khc* in the regulation of the apical Crb domain in developing photoreceptors. Since the rhabdomeres in developing pupal eyes grow along the distal-proximal axis, these phenotypes suggest that Khc is essential for the microtubule structures and apical membrane domains during the distal-proximal elongation of photoreceptors, but is dispensable for early eye development.

## Introduction

**Figure 1:**
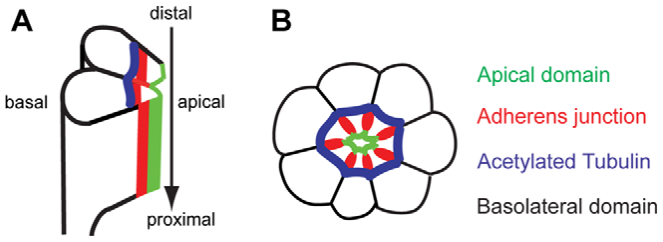
Acetylated microtubules of *Drosophila* pupal photoreceptors. (A) Side view of developing photoreceptors at mid-stage pupal development. The photoreceptors elongate along the distal-proximal axis (arrow). (B) Cross-section of mid-stage pupal photoreceptors. Apical domain (green) localizes apical to AJ (red) in the center of a photoreceptor cluster. E-cad localizes at AJ (red), which are more basal to the apical domain. The acetylated-tubulin (blue) localizes just basal to the AJs (red) and the basolateral domains (black) are more basal to both the AJ (red) and the acetylated-tubulin (blue).

The compound eye of *Drosophila* is made up of about 800 ommatidia, each of which is comprised of a cluster of eight elongated columnar photoreceptor cells covered by a thin layer of pigment cells [1], [2]. These clusters of 8 photoreceptor cells (R1–R8) are made in the eye disc epithelium during the third instar larval stage, before photoreceptor morphogenesis takes place. Along the length of each ommatidial column extends a light sensitive, tightly packed array of 60,000 microvilli called a rhabdomere [1], [2], [3]. At 37% pupal development (pd) stage, the apical region of each of the photoreceptor cells is involuted by 90°, reorienting the apical domains towards the center of the cluster (Fig. 1) [1], [2]. At this time, the apical membrane domain, having been localized at the center of the photoreceptor cluster, is now surrounded immediately by the AJs, followed by the basolateral domains (Fig. 1) [4], [5]. The formation of the rhabdomere from the apical surface of the photoreceptor cells begins at 55% pd and involves a series of complex cell-cell signaling interactions and the rapid expansion of the plasma membrane [1], [2], [3]. Because of the enormity of this extension and the rapidity with which it occurs, even small signaling defects can cause dramatic phenotypic consequences in the developing eye.

Genetic control of apical-basal cell polarity in the developing *Drosophila* eye is crucial for epithelial morphogenesis [4], [5], [6]. This control is obtained through a small number of evolutionarily conserved polarity proteins that play an important role in many versions of apical-basal cell polarization. These polarity proteins form two heterotrimeric cassettes: the Crb complex, consisting of Crb, Stardust (Sdt), and Patj, and the Par complex, consisting of Par-6, aPKC, and Bazooka (Baz) [7]. Baz, PAR-6, and aPKC form a complex that plays a key role in the polarization of many cell types and cell-polarity dependent organ morphogenesis. However, Baz localizaes at AJ, but Par-6/aPKC localize at the apical membrane domain of Crb/Sdt/Patj in photoreceptors [8], [9]. The Baz is excluded from the apical Par-6/aPKC domain in epithelia by aPKC phosphorylation, which disrupts the Baz/aPKC interaction [10], [11]. Removal of Baz from the Par-6/aPKC complex also requires the Crb complex, which prevents the Baz/PAR-6 interaction. In the absence of Crb or aPKC phosphorylation of Baz, mislocalized Baz recruits AJ components apically, leading to a loss of the apical domain and an expansion of lateral [10], [11].As the rhabdomeres begin to form at 55% pd, Crb complex proteins are positioned to the rhabdomere stalk, which connects the rhabdomere to the AJ. Meanwhile, the photoreceptor cells, including the rhabdomeres, undergo distal to proximal elongation, stretching from the distal region of the photoreceptor cells to the proximal base of the retina [1], [2]. Crb, though required for this extension, is not required for establishing apical-basal polarity in early eye development [4], [5].

Microtubules are essential components of cellular structure and function, playing critical roles in cell shape, polarity, and division. One of the ways in which microtubules perform these roles is by providing a means of transportation for various organelles and cellular cargo. This intracellular transportation occurs via microtubule-based motor proteins, which are capable of binding cargo and transporting the bound organelle or protein to its appropriate destination via ATP-driven mechanisms. Composed of α and β-tubulin heterodimers, microtubules display an intrinsic polarity due to the repeated head-to-tail linear protofilament associations of α-tubulin at the slowly growing minus ends and β-tubulin at the faster growing plus ends [12].

Recently, we identified the specific localization of stabilized/acetylated microtubule structures in developing *Drosophila* pupal photoreceptors (Fig. 1) [13]. It was also found that Spastin, a microtubule-severing AAA ATPase involved in constructing neuronal and non-centrosomal microtubule arrays, helps control the apical localization of Crb [13]. Since many membrane materials like Crb are targeted to the growing apical membranes during the massive growth of the rhabdomeres, it was hypothesized that there may be a microtubule-based motor protein such as Kinesin-1 that moves along the microtubules and targets the apical proteins to their specific regions of localization [13].

Kinesin-1, first identified in squid axoplasm, is a plus end-directed microtubule-based motor protein that is composed of two heavy chains and two light chains [14], [15], [16], [17]. Kinesin-1, along with other motor proteins, is essential in intracellular transport, whereby the motor protein binds cargo and generates movement coupled to ATP hydrolysis along cytoskeletal filaments [15]. In the case of Kinesin-1, microtubule motor activity is performed by Khc, which contains microtubule and ATP binding sites at its N-terminal head, whereas kinesin light chain (Klc) is used in most of Kinesin-1's cargo binding activity [14], [15], [17]. Interestingly, Khc can perform numerous functions in *klc* mutant *Drosophila* lines, suggesting Klc's dispensability in at least some contexts [17], [18], [19], [20]. Because the members of the Kinesin-1 subfamily of motor proteins are important in long range anterograde axonal transport and mutations in this family have been linked to neurodegenerative diseases like hereditary spastic paraplegia (HSP), which is most commonly associated with *spastin* mutations, Khc of Kinesin-1 serves as an excellent candidate for apical protein delivery along the newly identified microtubules of the developing *Drosophila* photoreceptor cells [13]. Since the microtubules in the *Drosophila* photoreceptor are oriented with their positive ends toward the apical domain with their minus ends near the nucleus [21], Kinesin-1's plus end-directed movement could be capable of delivering the necessary apical polarity proteins to their normal localizations. The Kinesin-1 motor protein is an ideal candidate to play a role in the sort of trafficking involved in *Drosophila* photoreceptor morphogenesis because of its plus end-directed movement, its high processivity, and its versatile heavy chain subunit. First, Kinesin-1 may be an excellent potential candidate for apical protein targeting in *Drosophila* photoreceptor morphogenesis due to the fact that the dramatic distal to proximal elongation that occurs in the mid-stages of pupal eye development would involve rapid microtubule growth that would only occur at the dynamic, rapidly extending plus ends, which are oriented toward the apical domain in *Drosophila* photoreceptors [21]. Second, Kinesin-1's high processivity, a product of Khc's dimerized motor domain, makes it highly capable of functioning in protein or microtubule transport during this demanding, highly coordinated phase of photoreceptor morphogenesis [16]. Third, the dimerized heavy chain subunits of Kinesin-1 are robust and versatile, responsible not only for its motor activity but also for some cases of cargo binding as well as the movement of rapidly growing, cell morphology-defining microtubules via Kinesin-mediated microtubule sliding [22].

We have therefore analyzed Khc's role in apical domain polarity protein targeting in developing pupal photoreceptors via the recently identified microtubule bundles in the pupal photoreceptors. We found that *khc* mutant photoreceptors display progressive defects in Crb localization at the apical membrane domain along the distal-proximal axis during rhabdomere elongation, without affecting early eye differentiation or pattern formation. Our data suggests that Khc is essential for apical membrane domain targeting during the exponential growth that occurs during rhabdomere elongation.

## Results

### Genetic Interactions Between *khc*, *crb*, and *spastin*

Previous studies have shown that Crb provides a positional cue for photoreceptor elongation [4], [5]. In *crb* mutants, the apical domain showed a progressive defect along the distal-proximal axis of elongation [4], [5]. Recently, a stabilized microtubule structure was discovered in pupal eyes and its modulation by Spastin was linked to apical polarity protein localization [13], [23]. Spastin functions as a microtubule modulator and its mutation in photoreceptors showed progressive distal-proximal apical domain defects similar to those observed in *crb* mutants. Furthermore, gain-of-function mutations in both *crb* and *spastin* caused apical domain expansions in developing pupal eyes [4], [13]. It is therefore hypothesized that Khc may have a role in a polarity protein trafficking along the stabilized microtubule tracks in pupal photoreceptors.

**Figure 2:**
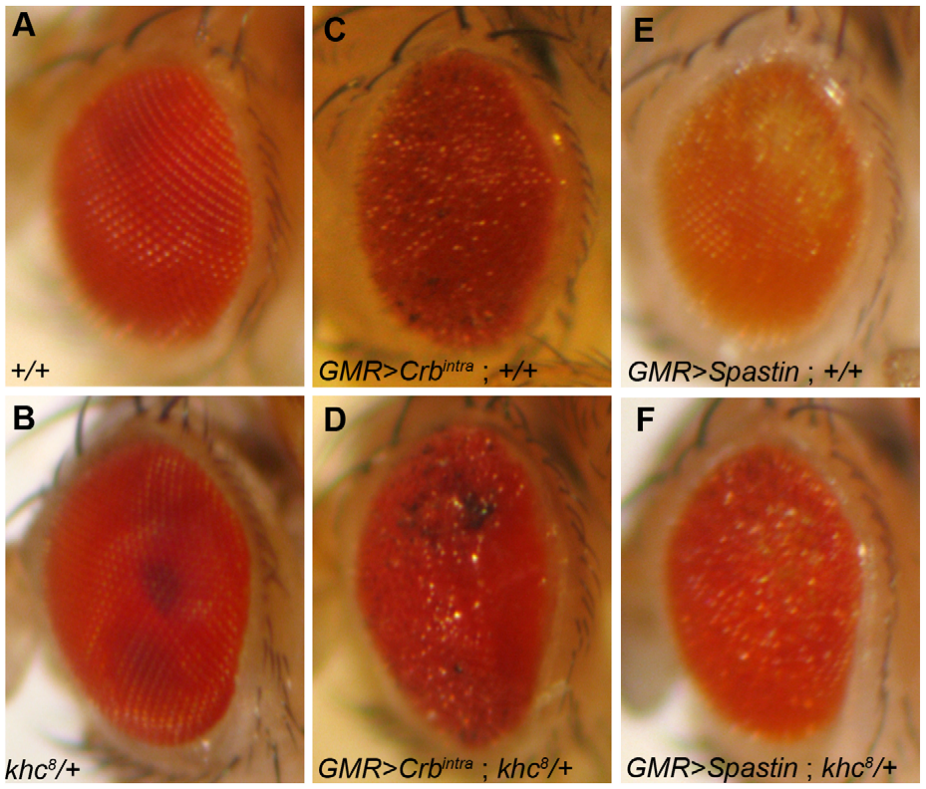
Genetic interactions of *khc*, *crb* and *spa* in *Drosophila eye*. (A–B) Adult eye phenotype of +/+ (A), *khc^8^/+* (B), *GMR>Crb^intra^*; +/+ (C), *GMR>Crb^intra^*; *khc^8^/+* (D), *GMR>Spastin*; +/+ (E), and *GMR>Spastin*; *khc^8^/+* (F).

We overexpressed the conserved Crb intracellular domain (*Crb^intra^*) [24] and *spastin* [25] using *GMR-Gal4* [26], which in both cases led to a roughening of the eye's external morphology (Fig. 2C and 2E) [27]. Using these genetically sensitized phenotypes, we performed tests to identify genetic interactions among *crb*, *spastin*, and *khc*. In these genetically sensitized conditions, the rough eye phenotypes were dominantly enhanced by reducing the level of *khc* in the *khc/+* heterozygous background (Fig. 2D and 2F), thus suggesting strong genetic interactions between *crb*-*khc* and *spastin-khc* in the *Drosophila* eye. The enhancements of the rough eye phenotypes in the *khc/+* heterozygous backgound were very consistent with 100% penetrance (n>100). This genetic interaction data strongly suggests that Khc may provide an additional cue for Crb and Spastin function in photoreceptor development because the rough-eye phenotypes caused by either c*rb* or s*pastin* overexpression were further enhanced by reduced *khc* gene dosage (*khc/+*).

The genetic interaction between *khc* and *crb* was established based upon the *crb* gain-of-function and *khc* loss-of-function combination. We utilized the classic and traditional genetic modifier test with the sensitized condition of *crb*. In this condition, the *khc/+* heterozygote dominantly enhanced the *crb* gain-of-function phenotype. We do not, therefore, treat the genetic interaction data as conclusive, but rather as preliminary data necessary to begin dissecting the role of *khc* in the Crb localization.

### Localization of Khc in Pupal Photoreceptors

**Figure 3:**
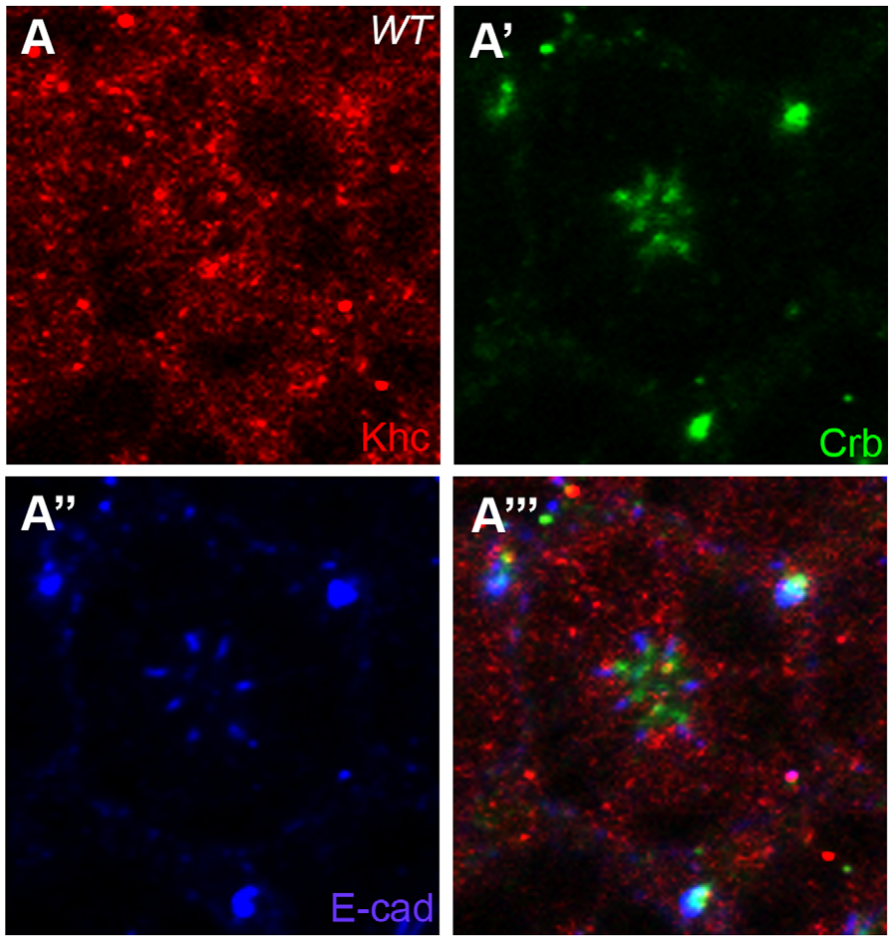
Localization of Khc in *Drosophila* pupal photoreceptors. Localization of Khc in mid-stage (50% pd) of *Drosophila* pupal photoreceptor. (A) Khc staining (red, A) was ubiquitous in the cytosolic regions of the photoreceptor and absent from the nuclear regions. Khc localization partially overlaps both the Crb (apical marker, green, A′) and E-cad (AJ marker, blue, A″) domains.

After finding genetic interactions among *khc*, *crb*, and *spastin*, it was then necessary to determine the localization of Khc in developing wild type photoreceptors. Staining with antibodies directed against Khc revealed a nearly ubiquitous and punctuated cytosolic distribution of Khc in mid-stage pupal photoreceptors (Fig. 3A). Some Khc localization partially overlaps both the Crb (Fig. 3A′) and AJ domains (Fig. 3A″) in the developing photoreceptors. Significantly, the only region of the photoreceptor cells lacking Khc was the nucleus (Fig. 3A), which is to be expected as the cellular organelles as well as the acetylated microtubule structures are located outside of the nucleus. The perinuclear localization of Khc is thus consistent with the localization of both Cnn and γ-tubulin, both of which are associated with the microtubule structures in the developing photoreceptor [23]. A similar ubiquitous cytoplasmic distribution of Khc was observed in early eye-discs of third-instar larvae (data now shown). The ubiquitous cytoplasmic distribution of Khc might represent its multiple functions in the photoreceptor cells.

### Khc is Dispensable in Early Eye Differentiation and Pattern Formation

To examine whether Khc is required for retinal differentiation in early-stage eye development, we generated mosaic eyes of a null mutation of *khc^8^* [16], [28], [29] using the FLP/FRT technique [30].

**Figure 4:**
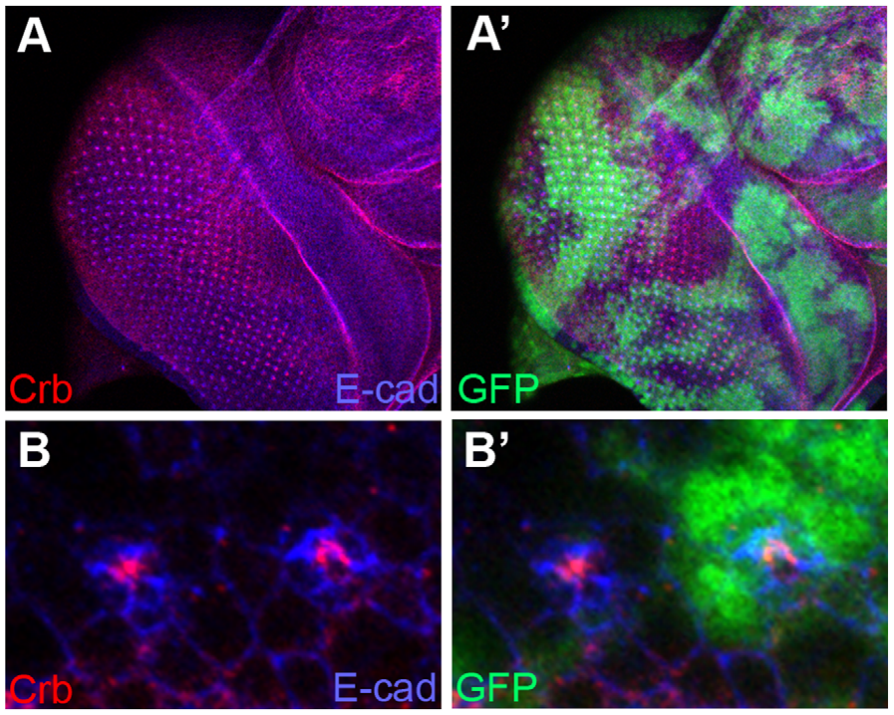
Khc is dispensable for early eye pattern formation. (A) A null mutant, *khc^8^*, was utilized to generate mutant clones in developing third-instar larval eye discs. Crb (apical marker, red) and E-cad (AJ maker, blue) showed little defects in third-instar larval eye discs. (B) A magnified view of (A). Mutant clones were marked by the absence of GFP (green).

In *khc* loss-of-function mutant clones in the eye discs of the third-instar larvae, the apical domains and AJs of photoreceptor precursor cells marked by Crb and E-cad, respectively, appeared to be normal (Fig. 4A). Furthermore, we did not find any obvious differentiation defects in the larval eye discs of *khc* mutants. Several other markers including neuronal and glial markers (Elav and Cut) showed little defects in the *khc* mutant clones of third-instar larval eye discs (data not shown). This data indicates that *khc* is dispensable for both the localization of cell junctions and for early differentiation in the eye.

### Khc is Required for Localization of the Apical Domain and Microtubules during Pupal Eye Development

**Figure 5:**
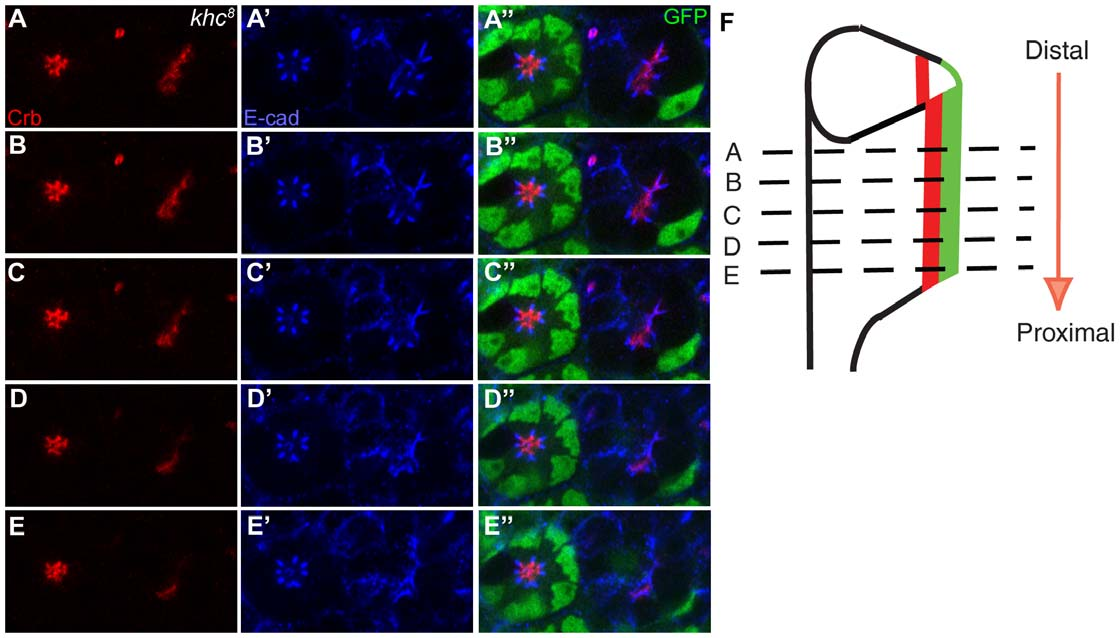
Khc is essential for photoreceptor morphogenesis. *khc^8^* mosaic clone in mid-stage (50% pd) pupal *Drosophila* photoreceptors. (A–E) *khc^8^* drosophila photoreceptors stained for Crb (apical marker, red), and E-cad (AJ marker, blue). (A′–E′) *khc^8^* mutant photoreceptors were marked by the absence of GFP (green). As the cross sections move more proximally (A–E) both the apical Crb domain (red) and the AJs (blue) show increasingly severe defects. Crb (red) is misshapen at the distal section (A, B) and almost absent at the proximal section (E) from the same pupal eye. (F) Developing pupal photoreceptors elongate from distal to proximal direction. Distal (A, B) and proximal (D, E) sections are marked by dashed-lines.

We examined whether Khc is required for photoreceptor morphogenesis in mid-stage pupal eye development. Although *khc* mutants did not show any defects in early larval eye discs (Fig. 4), progressive defects in both the Crb and AJ domains were observed along the distal-proximal axis in the mid-stage pupal eyes (Fig. 5), which undergoes elongation during this stage of the morphogenesis. The *khc* mutation caused a mild Crb mislocalization at the distal section (Fig. 5A and 5B), but caused almost complete loss of Crb at proximal sections of the same photoreceptor (Fig. 5E). Other apical markers of Sdt [31], [32], Patj [33], Par-6 [34] and aPKC [35], [36] showed the same progressive defect patterns in *khc* mutants (data not shown). It was noticed that the almost complete loss of Crb occurred even in the presence of the misshapen AJs (E-cad, Fig. 5E) in the proximal section of the *khc* mutant, which strongly indicates that the losses in the apical membrane domain represent the primary defect observed in the absence of Khc, rather than the defects that occurred in the misshapen AJs,. In *khc* mutants, other AJ markers of Baz [9], [37] and Armadillo (Arm, β-Catenin) [38] showed the same progressive misshapen defects as those observed in E-cad (data not shown). Furthermore, no cell polarity defects were detected in *khc* mutants since the apical Crb markers still localized at a more apical position relative to the AJ (E-cad) (Fig. 5). This data suggests that Khc may indeed facilitate the localization of apical membrane components including Crb, perhaps by utilizing the nearby microtubule tracks.

Because the rhabdomere grows from distal to proximal in developing pupal eyes (Fig. 5F, arrow), this mutant phenotype of *khc* strongly suggests that Khc is specifically required for apical membrane domain maintenance, including Crb localization, in distal to proximal rhabdomere growth during photoreceptor morphogenesis. This type of progressive apical defect along the distal-proximal axis of the rhabdomere was found in the case of c*rb*, *par-1* and *spastin* mutations [4], [5], [13], [39], [40]. Unlike photoreceptor cells, other eye accessory cells which surround the photoreceptors, such as cone cells, inter-ommatidial bristles, and pigment cells, were not affected in the *khc* mutant pupal eyes (data not shown). Thus, the observed defects in *khc* mutant eyes are not only specific to the developmental stage but also to the cell type.

**Figure 6:**
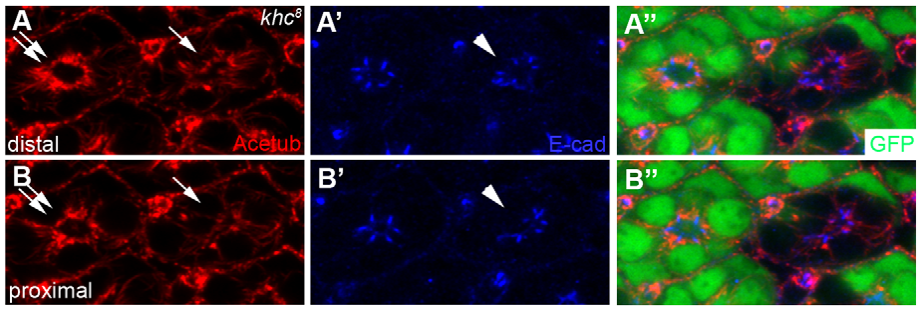
Khc is essential for acetylated microtubules and AJ localization. Stabilized microtubules were stained with Acetub (red), AJs with E-cad (blue), and wild type cells with GFP (green). (A-A″) Distal regions of *khc^8^* mutant photoreceptors, marked by the absence of GFP (green), display reduction and shrinkage of microtubules (arrow) compared to wild-type cells (double-arrow) and AJs (arrowhead) at the same location. (B-B″) Proximal regions of *khc^8^* mutant photoreceptors display more severe defects of acetylated microtubules (arrow) and AJs (arrowhead).

Furthermore, the differential defects along the distal-proximal axis were also identified in the stabilized/acetylated microtubules in *khc* mutants. The acetylated microtubules in *khc* mutant photoreceptors were reduced and shrunken from basal to apical position at the distal section (Fig. 6A, arrow), compared to those in wild-type cells (Fig. 6A, double-arrow), and even more reduced and shrunken at the proximal section of *khc* mutant cells (Fig. 6B, arrow). This data raised a possibility that the progressive Crb localization defects (Fig. 5) along the distal-proximal axis might be caused by the differential defects in acetylated microtubules along the distal-proximal axis during rhabdomere elongation. Because a massive amount of material trafficking is required during the rhabdomere elongation step, the lack of a functional Khc motor simply cannot provide sufficient amounts of apical Crb targeting to the growing membranes.

### Overexpression of *khc* Causes Crb and Stabilized Microtubule Reduction

The loss of function analysis of the *khc* mutation strongly indicates that Khc might play a role in the proper positioning of the Crb domain (Fig. 5) and the stabilized microtubules (Fig. 6) during photoreceptor morphogenesis. To further test this finding, a gain-of-function analysis of *khc* was conducted using an eye-specific Gal4 driver, *sevenless (sev)-Gal4*, in order to increase *khc* expression in the developing photoreceptors. The *sev-Gal4* driver targets expression in a subset of photoreceptors and cone cells of the *Drosophila* eye [42].

**Figure 7:**
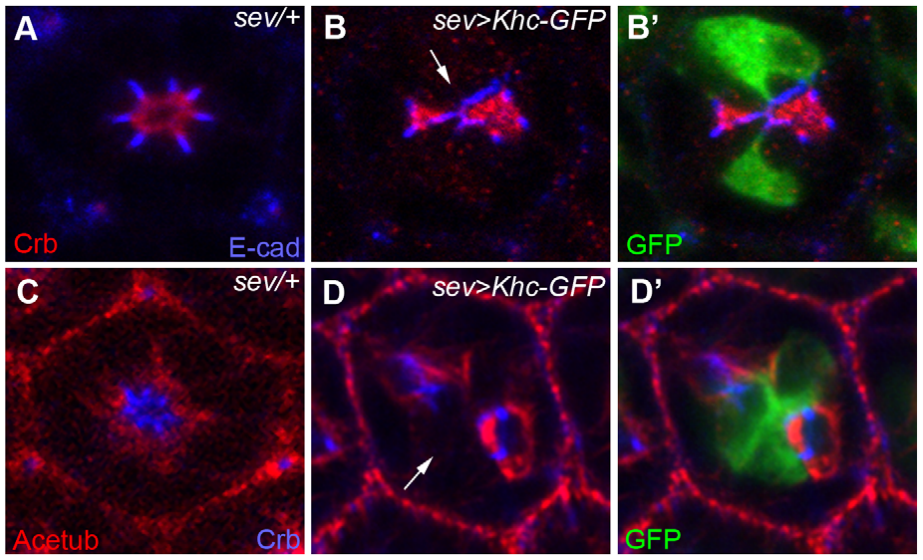
Overexpression of *Khc* causes severe reduction of acetylated microtubules and apical domain. Khc-GFP overexpression causes severe reduction of the apical membrane domain in developing pupal eyes (45% pd). (A, B) Localization of Crb (apical marker, red) and E-cad (AJ marker, blue) in the *sev-Gal4/+* control (A), and *Khc-GFP* overexpression by *sev-Gal4* (B). Khc-GFP was marked by GFP (green, B′). Khc-GFP caused a loss of Crb (red, arrow, B) and mislocalization of E-cad (blue). (C, D) Localization of Acetub (stabilized microtubule marker, red) and Crb (apical marker, blue) in the *sev-Gal4/+* control (C), and *sev>Khc-GFP* (D). *Gal4* caused the reduction of Acetub (red, arrow, D) and Crb (blue). Khc-GFP was marked by GFP (green, D′).

The previously established *UAS-Khc-GFP* [43] was used to test the effects of *khc* overexpression in photoreceptor morphogenesis. Overexpression of *khc* using *sev-Gal4* in mid-stage pupal photoreceptors resulted in the dramatic concurrent reduction of Crb (Fig. 7B and 7D) and the stabilized microtubules (Fig. 7D), while the misshapen AJ defects were relatively less severe (Fig. 7B) compared to those observed in Crb. This data indicates that the overexpression of *khc* causes the disruption/reduction of both Crb and the stabilized microtubules in developing pupal photoreceptors. The reduced Crb and misshapen AJ phenotypes observed in the *khc* gain-of-function mutation (Fig. 7) are similar to those observed in the *khc* loss-of-function mutation (Fig. 5). The similarity between these phenotypes suggests that there might be a dominant-negative effect in the *khc* gain-of-funciton mutation. In the eye discs of the third-instar larvae of *sev>khc*, there was no obvious differentiation defects (data not shown), which is similar to the case of *khc* mutant (Fig. 4).

Additionally, there were cell non-autonomous effects of the Khc overexpression in Crb/AJ (Fig. 7B) and acetylated tubules (Fig. 7D). However, this non-autonmous effects were not observed in *khc* mutants (Figs. 5 and 6). This finding indicates that Khc overexpression can elicit non-autonomous effects in other cells which do not express Khc, opening up the possibility that Khc could affect neighboring cells through effects on cell junctions. However, other potentially unnatural Khc targeting of Khc could not be excluded.

This *khc* gain-of-function data strongly suggests that Khc specifically controls the apical membrane domain and the stabilized microtubules during pupal photoreceptor morphogenesis. These effects of Khc on Crb and the stabilized microtubules were also seen in the *khc* loss-of-function mutants, in which the defects were progressively severe along the distal to proximal elongation axis (Figs. 5 and 6). These results strongly suggest that Khc facilitates apical protein targeting while simultaneously promoting the more basal stabilized microtubule structures during the massive amounts of growth which occur during rhabdomere elongation. This might indicate that there is a synergistic effect between the trafficking road and the apical polarity protein delivered by the microtubule-dependent motor.

## Discussion

Recently we have established a link between the maintenance and modulation of stabilized microtubules and Spastin function in mid-stage pupae, with *spastin* mutations causing a microtubule defects and a progressive loss of Crb along the distal-proximal axis of the elongating photoreceptors [13]. This progressive defect was initially found in the *crb* mutation [4], [5]. These phenotypes may be due to the massive requirements of the apical domains during the rhabdomere elongation stage of developing photoreceptors, which may be dependent upon the microtubule modulating function of Spastin and Crb as an apical positional cue. The genetic interactions between *khc*, *crb*, and *spastin* described in the present study further confirm these relationships. This genetic data therefore suggests that Khc may play a role for the apical domain modulating activities of Crb and Spastin. However, their genetic interactions might not be ubiquitous or direct, because the *khc* mutant do not show any defects in Crb during early larval eye development.

In confirmation of this potential role for Khc, it is also discovered that the *khc* null mutation leads to the progressive, distal to proximal mislocalization of Crb, with increasingly severe mislocalizations occurring the further proximally the photoreceptor extends (Fig. 5). Both the apical Crb domain and the more basal AJs were basally misshapen (Fig. 7), with greater deformities occurring in the former, thus indicating that the normal functioning these domains and the proper localization of their respective polarity proteins is contingent in part upon the proper functioning of Khc. The function of Khc with respect to Crb was further supported by the gain-of-function analysis, which disrupts the apical Crb domain (Fig. 7).

Based on the progressive loss of the apical domains of the pupal photoreceptors during rhabdomere elongation (Fig. 5), it is proposed that Khc might specifically control the proper localization of the apical Crb domain. This apical domain-specific function of Khc is based on the following data: (i) the potential role of Khc as a microtubule-based motor for the apically-targeted proteins, (ii) the *khc* mutation caused apical domain defects (Fig. 5), and (iii) overexpression of *khc* caused apical domain disruptions (Fig. 7). However, another possibility cannot yet be excluded, namely, the direct modulation of the microtubules by Khc [22], [41] during the rhabdomere elongation. The progressive defects observed in the stabilized microtubule structures during rhabdomere elongation in the *khc* mutants (Fig. 6) might affect the potential trafficking road on which apical proteins are transported. Nevertheless, these two possibilities are not necessarily mutually exclusive.

The *khc* mutation resulted in a partial loss of Crb and AJ domains in addition to a progressive distal-to-proximal disruption of acetylated microtubules which localize basal to the Crb and AJ domains. Significantly, the partial losses of both Crb and the AJs occurred most conspicuously in regions that had also experienced a concurrent loss of stabilized microtubule structures (Fig. 6). This finding further confirms the potential role of Khc in proper microtubule modulation along the rapidly expanding pupal photoreceptor and the subsequent use of these microtubule bundles in protein transport. This may be accomplished via Khc's role in Kinesin-l-mediated microtubule sliding [5], which drives the transportation of short microtubules and their “piggybacking” cargo toward the terminal processes of expanding regions of cells, thus driving changes in cell shape, as in the drastic distal to proximal expansion that occurs during *Drosophila* photoreceptor morphogenesis.

A theoretical model for the establishment of initial cell polarity that emerges from these findings involves a feedback loop which has a regulatory component and a cytoskeletal component. Both components modify each other's behavior to form a feedback loop that is critical for generating and maintaining cell polarity in the growing rhabdomere [44], [45], [46]. It is possible that the microtubules function as the cytoskeletal component and the apical polarity proteins function as the regulatory component in our model. If Khc is absent, this positive feedback loop may be disrupted. This model may explain why each of the two components displayed the progressive *crb* mutant-like phenotypes in *khc* mutants. However, this possibility of mutual interdependence between the microtubule and polarity proteins needs to be studied further.

Because we have identified pupal eye defects in *khc* mutants, we would expect to see similar disruptions in adult photoreceptors in the absence of the functional Khc. In fact, the adult eye defects in *khc* mutants have been previously described [3], with adult mutant eyes showing a reduction in rhabdomere numbers and/or split or bundled rhabdomeres [3], which were also observed in the adult eyes of *crb* mutants which showed rhabdomere elongation defects similar to those observed in *khc* mutants [4].

A recent study on the role of the Kinesin-2 (Klp64D, Kif3A) motor protein in developing *Drosophila* photoreceptors found that Kinesin-2 is essential for Baz and Armadillo (Arm, an AJ marker) targeting to the AJ beginning in early differentiation, as well as for photoreceptor cell survival [47]. Thus, Kinesin-2 appears to be responsible for the initial targeting of Baz, a key nodal component for other cell polarity proteins that is responsible for the localization of the apical proteins of the Crb and Par complexes in photoreceptor [8], [9], [39], [40]. Mutations in *kinesin-2* therefore led to severe defects, including a decrease in cell viability and improper nuclear positioning in differentiating photoreceptors [47]. In the *khc* mutant, however, the initial targeting of all cell polarity proteins was not affected (Fig. 4), with progressive defects (Fig. 5) gradually occurring during the later pupal morphogenesis stage. Therefore, Kinesin-1's role in developing photoreceptors does not include the initial targeting of cell polarity proteins like Baz, as its function appears to be mostly restricted to the later stages of morphogenesis. These contrasting effects in *kinesin-1* and *kinesin-2* mutant pupal photoreceptors highlight the varying roles that microtubule-based motor proteins must perform in coordinated fashion in order to ensure the proper polarization and subsequent morphogenesis of *Drosophila* photoreceptor cells.

## Materials and Methods

### Genetics

All *Drosophila* strains were grown and maintained at room temperature. Mitotic recombination was induced by using the FLP/FRT method for clonal analysis [30]. *Khc^8^* is a null allele with a nonsense mutation that truncates the protein at amino acid 210. The resulting protein is unstable, resulting in a severe phenotype and a lack of detectable Khc [16]. *khc^8^* null mutants have been completely rescued by the *P*(*khc^+^*) transgene [28], [29]. *khc^8^* mutant clones [16] were produced by eye-specific expression of Flp (ey-Flp) in *y w ey-Flp/+*; *FRT42D khc^8^/FRT42D Ubi-GFP*. *UAS-Spastin* [25] and *UAS-Crb^intra^* [24] were recombined on the same chromosome with *GMR-Gal4* for a genetic modifier test. Overexpression of Khc was induced by crossing *UAS-Khc-GFP* with *sev-GAL4* at room temperature (22°C) [26]. *khc8*, *UAS-Khc-GFP*, and *sev-Gal4* were obtained from the Bloomington Stock Center at Indiana University.

### Immunohistochemistry

The following primary antibodies were used: mouse anti-acetylated tubulin (Sigma), 1∶1000; mouse anti-Arm (N2 7A1, DSHB) [38], 1∶10; rabbit anti-Baz [37], 1∶1000; rat anti-E-cadherin (Dcad2, DSHB) [48], 1∶10; mouse anti-Cut (2B10, DSHB) [49], 1∶10; mouse anti-Khc (Suk 4, DSHB) [50], 1∶25; mouse anti-Crb (Cq4, DSHB) [51], 1∶10; rat anti-Crb [33], 1∶400; rat anti-Elav (DSHB) [52], 1∶25; sheep anti-GFP (Biogenesis or Serotec), 1∶100; rabbit anti-Par-6 [34], 1∶500; rabbit anti-PKCzeta (Santa Cruz), 1∶500; rabbit anti-Sdt, 1∶500 [31], [32]. A 15 minute acetone or methanol treatment was performed after fixation for mouse or rat anti-Crb staining, respectively. Secondary antibodies obtained from Jackson Laboratories conjugated with Cy3, Cy5, or FITC. Fluorescent immunostaining and confocal analysis of pupal eyes was carried out as reported [9], [13], [23], [27], [39], [53]. Fluorescent images were acquired on an Olympus FV1000 confocal microscope. Image analysis and quantification were performed using ImageJ (NIH) and Adobe Photoshop (Adobe Systems Incorporated).
